# Supplementary material for: Pharmacokinetics of Tildipirosin in Ewes after Intravenous, Intramuscular and Subcutaneous Administration
Source: Animals (Basel). 2020 Aug 1;10(8):1332. doi: 10.3390/ani10081332 (PMC7460420; doi:10.3390/ani10081332)
Supplement: Supplementary file 1 [file animals-10-01332-s001.zip › animals-868228-supplementary.docx]

Supplementary Materials: Pharmacokinetics of Tildipirosin in Ewes After Intravenous, Intramuscular and Subcutaneous Administration.

Juan Sebastián Galecio ^1,2,^*, Elisa Escudero ^1^, José Joaquín Ceron ^3^, Giuseppe Crescenzo ^4^ and Pedro Marin ^1,^*.

^1^ Department of Pharmacology, Faculty of Veterinary Medicine, University of Murcia, 30100 Murcia, Spain. [escudero@um.es](mailto:escudero@um.es) (E.E.); [pmarin@um.es](mailto:pmarin@um.es) (P.M.)

^2^ Escuela de Medicina Veterinaria. Colegio de Ciencias de la Salud. Universidad San Francisco de Quito. EC 170157. Cumbayá, Ecuador; [jgalecio@usfq.edu.e](mailto:jgalecio@usfq.edu.e)c

^3^ Interdisciplinary Laboratory of Clinical Pathology, Interlab–UMU, University of Murcia, 30100 Murcia, Spain. [jjceron@um.es](mailto:jjceron@um.es)

^4^ Department of Veterinary Medicine, University of Bari Aldo Moro, 70010-Valenzano, BA, Italy. [giuseppe.crescenzo@uniba.it](mailto:giuseppe.crescenzo@uniba.it)

***** Correspondence: [jgalecio@usfq.edu.e](mailto:jgalecio@usfq.edu.e)c; Tel.: +593-2-2971700. (J.S.G). [pmarin@um.es](mailto:pmarin@um.es); Tel.: +34-660271337 (P.M).

Received: 30 June 2020; Accepted: 29 July; Published: date

**Table A1.** Mean ± SEM haptoglobin, CK–MB, CK and troponin concentrations in plasma of ewes after subcutaneous, intramuscular and intravenous of tildipirosin (*n* = 6).

|  |  |  | | **Time (days)** | | | | | | | | | | | | | | | | | | | | | |
| --- | --- | --- | --- | --- | --- | --- | --- | --- | --- | --- | --- | --- | --- | --- | --- | --- | --- | --- | --- | --- | --- | --- | --- | --- | --- |
| **Parameter (units)** | **Administration** |  | **Basal** | | |  | **0.5** | | |  | **1** | | |  | **2** | | |  | **3** | | |  | **4** | | |
| Haptoglobin (g/L) | SC |  | 0.112 | ± | 0.066 |  | 0.091 | ± | 0.056 |  | 0.075 | ± | 0.071 |  | 0.148 | ± | 0.053 |  | 0.072 | ± | 0.048 |  | 0.104 | ± | 0.056 |
| Haptoglobin (g/L) | IM |  | 0.113 | ± | 0.049 |  | 0.075 | ± | 0.035 |  | 0.062 | ± | 0.029 |  | 0.097 | ± | 0.027 |  | 0.069 | ± | 0.034 |  | 0.074 | ± | 0.044 |
| CK  (UI/L)* | IM |  | 101.2 | ± | 24.4 |  | 1610.8 | ± | 544.3 | a | 1354.8 | ± | 397.7 | a | 587.1 | ± | 95.2 |  | 190.4 | ± | 152.0 |  | 127.1 | ± | 164.7 |
| CK–MB (UI/L) | IV |  | 176.6 | ± | 59.0 |  | 143.8 | ± | 14.4 |  | 126.3 | ± | 16.3 |  | 152.2 | ± | 19.4 |  | 172.2 | ± | 32.5 |  | 136.5 | ± | 6.4 |
| Troponin (ng/mL) | IV |  | 0.110 | ± | 0.075 |  | 0.114 | ± | 0.061 |  | 0.108 | ± | 0.068 |  | 0.106 | ± | 0.060 |  | 0.109 | ± | 0.072 |  | 0.104 | ± | 0.066 |

a Indicate significant differences between basal and days after administration of tildipirosin. *Harmonic mean. CK: Creatine kinase. CK–MB: Creatine Kinase cardiac isoenzyme
